# Variable Clinical Presentations and Renal Outcome in Neonates With Autosomal Recessive Polycystic Kidney Disease

**DOI:** 10.7759/cureus.59993

**Published:** 2024-05-09

**Authors:** Ei Khin, Divya Ramdas

**Affiliations:** 1 Pediatric Nephrology, Texas Tech University Health Sciences Center El Paso, Paul L. Foster School of Medicine, El Paso, USA; 2 Pediatric Nephrology, El Paso Children's Hospital, El Paso, USA; 3 Pediatrics, Texas Tech University Health Sciences Center El Paso, Paul L. Foster School of Medicine, El Paso, USA; 4 Pediatrics, El Paso Children's Hospital, El Paso, USA

**Keywords:** cilia-related cystic kidney disease, nephronopthisis, jourbet syndrome, autosomal dominant polycystic kidney disease, autosomal recessive polycystic kidney disease, pediatric cystic kidney disease

## Abstract

Autosomal recessive polycystic kidney disease (ARPKD) is caused by a mutation in the polycystic kidney and hepatic disease-1 (PKHD1) gene and is an important inherited cause of chronic kidney disease in children. The most typical presentations in neonates are massively enlarged kidneys with variable echogenicity, multiple small cysts, and congenital hepatic fibrosis. Potter sequence with pulmonary hypoplasia can present due to oligohydramnios. Severe pulmonary hypoplasia can lead to respiratory insufficiency and perinatal death. Some affected children can develop end-stage renal disease in early childhood or adolescence. Here, we report the clinical presentations, management, and renal outcomes of three neonatal cases of ARPKD from our center.

## Introduction

Autosomal recessive polycystic kidney disease (ARPKD; MIM#263200) is a rare inherited cause of end-stage renal disease (ESRD) in children, and its incidence is 1:20,000. It is caused by mutations of the PKHD1 gene (polycystic kidney and hepatic disease-1, also known as ciliary IPT domain containing fibrocystin/polyductin) on chromosome 6p12 [1].

ARPKD has been known as “infantile” polycystic kidney disease, and it primarily affects only two organs: the kidney (polycystic kidneys) and the liver (congenital hepatic fibrosis) [1]. ARPKD cysts exclusively collect tubules of origin and microcysts that cannot be visualized on gross examination. Congenital hepatic fibrosis and Caroli’s disease are typical extra-renal manifestations of ARPKD. Caroli’s disease is a biliary ductal plate malformation characterized by periportal fibrosis and non-obstructive bile duct proliferation with normal hepatocyte structure and function [1,2].

Renal insufficiency may begin in utero, which can lead to early abortion or oligohydramnios. ARPKD patients are diagnosed prenatally in the latter stages of pregnancy or at birth with evidence of enlarged hyperechogenic kidneys. In the presence of oligo- or anhydramnios due to renal function impairment, the affected fetus may develop pulmonary hypoplasia and Potter’s sequence, a characteristic facies with contracted limbs with club feet [2].

Cystic kidney diseases are clinically and genetically heterogeneous. ARPKD is one of the cilia-related disorders characterized by fibrocystic hepatorenal changes and variable systemic manifestation [3]. Due to a growing number of cilia-related cystic kidney diseases, distinguishing ARPKD from other cystic kidney diseases is challenging. Molecular diagnostic analysis is the gold standard for diagnosing ARPKD [4].

Despite dramatic advances in neonatal care, ARPKD is still associated with significant morbidity and mortality [3,5]. Up to 30% of patients die in the perinatal period, and some affected children reach ESRD that requires initiating dialysis or kidney transplant in infancy, early childhood, or adolescence [6].

In this paper, we reported three neonatal ARPKD cases and discussed the challenges in diagnosis and clinical management.

The abstract was previously presented as a poster at the American Society of Nephrology Kidney Week 2023 in Philadelphia, USA, in November 2023.

## Case presentation

Here, we present three cases of neonatal ARPKD with variabilities in clinical presentation and renal outcome.

Case 1

The patient is a two-day-old male neonate born at 33 weeks gestation, with a birth weight of 2053 grams, to a 33-year-old G6P1 (gravidity and parity) mother via C-section. He was prenatally diagnosed with oligohydramnios, bilateral enlarged ventricles in the brain, and bilateral cystic kidney disease. Toxoplasmosis, rubella, cytomegalovirus, and herpes were negative. There is no family history of cystic kidney disease. The mother’s previous pregnancies resulted in one spontaneous abortion at 10 weeks, two ectopic pregnancies at five weeks, a therapeutic abortion at 10 weeks, and an emergency C-section at 35 weeks due to severe pre-eclampsia.

In the neonatal ICU, the patient was intubated on a ventilator for one day and stable on room air by day six of life. During the initial 22 hours of life in the neonatal ICU, the patient developed oliguria that required to be given furosemide in a total of eight doses over three days. Urine output was improved by day four of life without requiring furosemide anymore.

The patient had no acute distress, edema, fluid overload, or abdominal mass on physical examination. There were no other abnormal findings, such as bone deformities and polydactyly. Laboratory results on day seven of life showed serum creatinine (Cr) of 0.7 mg/dL (normal value, 0.2-0.8 mg/dL), blood urea nitrogen (BUN) of 7 mg/dL (normal value, 7-20 mg/dL), sodium of 131 mmol/L ( normal value, 135-145 mmol/L), and potassium of 4.7 mmol/L (normal value, 3.5-5.5 mmol/L). Urinalysis demonstrated no blood or protein in the urine.

**Figure 1:**
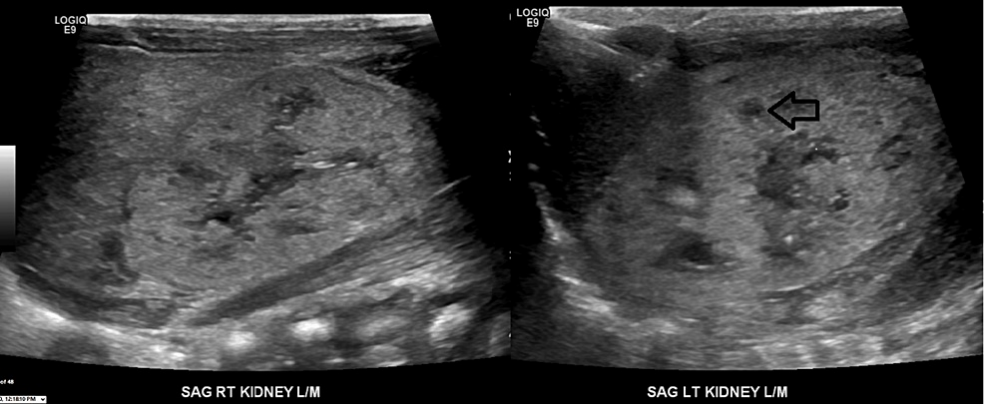
Echogenic kidney and small cyst (black arrow) in renal ultrasound of the left (LT) kidney

Post-natal imaging of kidneys showed bilateral dysplastic kidneys with numerous cysts but no enlargement of overall kidney size (Figure 1).

The urinary tract showed no abnormalities. Head ultrasound showed enlargement of lateral ventricles. Magnetic resonance imaging (MRI) of the brain showed ventriculomegaly without obstructive hydrocephalus. Abdominal ultrasound showed hyperechogenic liver without focal abnormality, normal-appearing gallbladder, and common bile duct. The echocardiogram showed a small atrial septal defect (ASD) and mild left-sided peripheral pulmonary stenosis.

He developed severe hypertension starting at eight weeks of age, requiring multiple antihypertensive medications, and a ventriculoperitoneal shunt was placed at seven months of age. His serum creatinine was 0.3 mg/dL at one year of age.

Case 2

This patient is a three-day-old male neonate born at 31 weeks, with a birth weight of 1500 grams, to a 29-year-old G1P1 mother via normal spontaneous vaginal delivery. A prenatal 20-week ultrasound showed possible polycystic kidney disease, and at 30 weeks gestation, the scan showed low amniotic fluid. There is no known history of cystic kidney disease in parents.

The patient required oscillator ventilation and transitioned to a conventional ventilator by day three of life and continuous positive airway pressure (CPAP) by day 15 of life. 

During the physical exam, the patient showed no signs of edema. On day seven of life, laboratory results showed serum Cr of 0.8 mg/dL, BUN of 21 mg/dL, sodium of 145 mmol/L, and potassium of 3.5 mmol/L. Renal ultrasound after birth showed normal-sized kidneys, with the right measuring 4.2 cm and the left measuring 4.9 cm; however, it showed bilateral mild pelviectasis and cysts, mostly on the left. The head ultrasound was unremarkable. A liver ultrasound showed multiple hepatic cysts, normal flow and direction of the portal vein, and a normal common bile duct. The echocardiogram showed a large PDA with bidirectional flow but no valvular cardiac abnormalities.

By eight weeks of life, the patient had significant hypertension that required treatment with Lisinopril, amlodipine, and metoprolol. His serum creatinine was 1 mg/dL at one year of age.

He developed ESRD at the age of four and received a pre-emptive living unrelated kidney transplant.

Case 3

The patient is a two-day-old female born at 36 weeks gestation, with a birth weight of 2600 grams, to a 32-year-old G1P1 mother born via C-section. Pregnancy was complicated by severe oligohydramnios at week 31, along with renal ultrasound showing bilateral enlarged echogenic kidneys. Family history is significant for the diagnosis of ARPKD in the patient’s cousin.

She was found to have pulmonary hypoplasia, requiring oscillator ventilation for five days. She subsequently had minimal urine output and developed fluid overload at three weeks of age despite intravenous furosemide treatment.

On initial physical exam, kidneys were palpable bilaterally, and mild hepatosplenomegaly was noted. On day seven of life, laboratory results showed serum Cr of 1.7 mg/dL (maximum serum Cr, 2.5 mg/dL), BUN of 47 mg/dL, sodium of 143 mmol/L, and potassium of 3.6 mmol/L.

**Figure 2:**
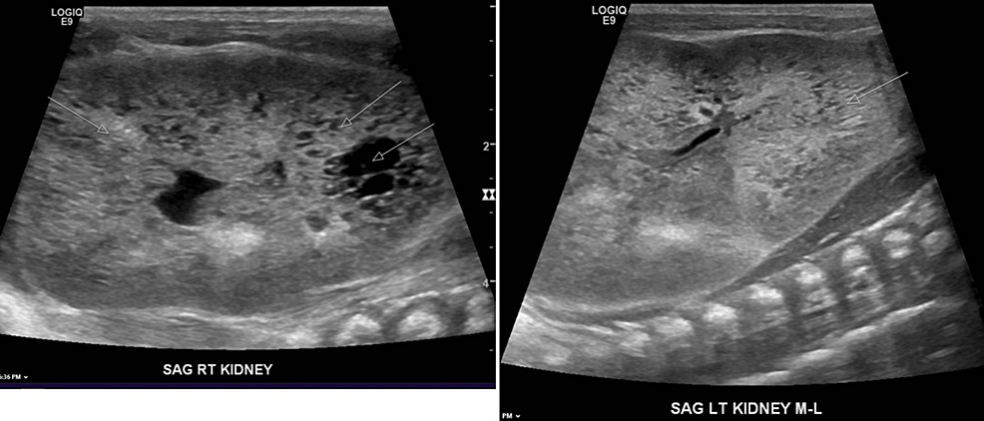
Enlarged echogenic right (RT) kidney showing multiple cysts (upper white arrows) and a large cyst (lower white arrow) and left (LT) kidney showing dilatation of collecting ducts (white arrow)

Renal ultrasound is remarkable for enlarged kidneys greater than 9 cm bilaterally with increased hyperechogenicity, suggesting multiple tiny cysts present bilaterally (Figure 2).

Initial chest X-ray showed low lung volumes and small right and left pneumothoraces. The head ultrasound was unremarkable. Abdomen ultrasound showed normal liver, no fibrosis, and normal flow of the portal vein.

The patient underwent a left nephrectomy and started peritoneal dialysis at one month of life. Subsequently, a right nephrectomy was done at three months of life.

Clinical and laboratory findings of cases are shown in Table 1.

**Table 1:** Clinical and laboratory findings of three neonatal cases with ARPKD

	Case 1	Case 2	Case 3
Gestational age (weeks)	33	31	36
Birth weight (grams)	2053	1500	2600
Oligohydramnios	Yes	Yes	Yes
Pulmonary hypoplasia	None	Yes	Yes
Renal ultrasound findings	Normal-sized kidneys and bilateral kidney cysts	Enlarged kidneys with pelviectasis and cysts	Very enlarged echogenic kidneys >9 cm bilaterally with multiple tiny cysts
Extra-renal manifestations	Ventriculomegaly and colpocephaly	Hepatic cysts	Pulmonary hypoplasia
Serum creatinine at day 7 of life (mg/dL) (reference range, 0.2-0.8 mg/dL)	0.7	0.8	1.7
Serum creatinine at day 28 of life (mg/dL)	0.5	0.6	2.3
Initiation of dialysis before 28 days (yes/no)	No	No	Yes
Serum creatinine at 1 year of age (mg/dL)	0.3	1	N/A

## Discussion

ARPKD is usually diagnosed around the perinatal period. The diagnosis is highly suspected in the setting of bilateral enlarged echogenic kidneys with or without oligo-anhydramnios and liver abnormalities (congenital hepatic fibrosis). According to the modified Zerres criteria, the clinical diagnosis of ARPKD is suspected when typical kidney imaging findings in addition to one or more of the following: (1) typical liver imaging, clinical, or laboratory findings consistent with congenital hepatic fibrosis; (2) liver pathology showing a biliary ductal plate malformation; (3) negative standard ultrasound findings in both parents or characteristic findings on high-resolution ultrasonography; and (4) diagnosis of ARPKD in an affected sibling by either pathology or genetics [7,8]

In terms of diagnosis in our case-cohort, the clinical diagnosis of ARPKD in case 1 was challenging. Case 1 presented with bilateral cystic kidney diseases, ventriculomegaly, and maternal history of oligohydramnios. No specific diagnosis of ARPKD in the affected siblings was made, although ARPKD was highly suspected due to the patient’s mother having had multiple miscarriages and a stillbirth history. Ventriculomegaly can be an incidental finding in preterm neonates. The decision was made to do an MRI of the brain. Joubert syndrome, one of the cilia-related cystic kidney diseases with cerebellar vermis hypoplasia, was ruled out with a brain MRI [9]. Due to unclear clinical presentations, genetic testing was done in the neonatal period to confirm the diagnosis. The genetic test result showed two different heterozygous pathological variants in the PKHD1 gene at c.5221G>A (p.Val1741Met) likely pathogenic and c.390+1G>T (p.?) pathogenic.

In terms of other clinical manifestations, hypertension management can be very challenging in ARPKD patients and requires the use of multiple anti-hypertensive medications. The prevalence of systemic hypertension in ARPKD patients from various cohorts is 33-75% [5,10-12], and the mechanisms are poorly understood. Limited animal and human studies suggested that dysregulation of the collecting duct epithelial sodium channel due to impaired urinary dilution is associated with fluid retention and increased activity of the intra-renal renin-angiotensin-aldosterone system without elevation in serum angiotensin I and II levels [13,14]. Angiotensin-converting enzyme inhibitors (ACEI) and angiotensin receptor blockers (ARB) are the mainstay agents. Expert opinion from consensus guidelines did not recommend using a combination of ACEI and ARB due to the increased risk of side effects without clear added benefit [2].

Urine output and serum electrolytes should be monitored in these neonates, especially those with a history of oligo-anhydramnios and high serum creatinine. The incidence of hyponatremia is 6-26% [5,7], and the mechanism is impaired urinary dilution, which can be coupled with a low osmotic diet [2]. 

Children with ARPKD are at higher risk of developing UTI, with reported rates of 20-50% in various cohorts due to urinary stasis in cystic, dilated collecting ducts [5,12,15].

From a renal outcomes perspective, although case 1 had challenges related to the control of hypertension with multiple antihypertensive medications, his kidney function was normal until two years of age. Case 2 developed chronic kidney disease stage 3 at two years of age and ESRD at four years of age. Case 3 presented with a severe manifestation of kidney impairment since her serum creatinine reached 2.5 mg/dL in the neonatal period. Due to her bilateral enlarged kidneys that can compromise respiratory and nutrition support, she underwent bilateral nephrectomies, G-tube placement, and peritoneal dialysis at two months of age. The optimal approach for unilateral or bilateral nephrectomies is not well established in infants due to limited case reports and case series [16-18]. Our unique cases highlighted the variable renal outcomes of ARPKD cases that were presented in the neonatal period.

Mutations of genes that cause nephronophthisis, Joubert, Bardet-Biedal, and ciliopathies-related disorders and HNF1ß mutation can mimic polycystic kidney diseases [19]. Due to the broad phenotypic and genetic heterogeneity of cystic kidney disease, genetic diagnosis is essential for the clinical management of the patients and helpful for families. Next-generation sequencing allows the analysis of large groups of genes at a relatively low cost and has become the mainstay for diagnosis due to the increasing number of genes [4]. Cases 2 and 3 had their diagnosis later confirmed with genetic testing.

In various cohorts, ARPKD patients invariably present with portal hypertension, esophageal varices, hypersplenism, and thrombocytopenia due to progressive portal tract fibrosis and cholangitis. In our case-cohort, the above-mentioned consequences were not noted in the neonatal period. A subset of ARPKD patients can have a predominant liver phenotype with few or no manifestations of kidney disease [20].

## Conclusions

ARPKD is one of the inherited causes of chronic kidney disease in children. Our case 3 typically presented bilaterally enlarged echogenic kidneys and had a serum creatinine of 2.5 mg/dL in the neonatal period. Clinicians should be aware of variable presentations of ARPKD cases and renal outcomes. Some may require aggressive management with bilateral nephrectomies and dialysis in the neonatal or infantile period. Clinical management is directed by multidisciplinary care teams consisting of perinatologists, neonatologists, nephrologists, gastroenterologists, geneticists, pediatric surgeons, nutritionists, and behavioral specialists to coordinate patient care from the perinatal period to adulthood. Due to the growing number of cilia-related cystic kidney disease, diagnosis can be confirmed with genetic testing.
